# Method of Iterative Determination of the Polarized Area of Steel Reinforcement in Concrete Applied in the EIS Measurements

**DOI:** 10.3390/ma15093274

**Published:** 2022-05-03

**Authors:** Mariusz Jaśniok

**Affiliations:** Faculty of Civil Engineering, Silesian University of Technology, 5 Akademicka St., 44-100 Gliwice, Poland; mariusz.jasniok@polsl.pl

**Keywords:** concrete structures, reinforcing steel, corrosion, NDT, modelling, equivalent electrical circuits, electrochemical impedance spectroscopy, EIS, polarization area

## Abstract

A new method is proposed for determining the test surface of steel rebar in concrete during polarization measurements of corrosion rate of reinforcement using the method of Electrochemical Impedance Spectroscopy. The methodology was based on the original *3D model* of the steel-concrete system, in which traditional equivalent electrical systems were coupled with factors that accounted for the complex geometry of the test reinforced concrete element. The developed method worked with a rectangular counter electrode without a guard ring assist, during an individual impedance measurement. The impact of the counter electrode size on the impedance spectra was verified in the first stage by tests conducted with ten types of counter electrodes. The obtained results in the form of empirical spectra were represented by theoretical spectra using the *3D model* and the matching degrees were within a range of 0.96–1.73 at the expected level of 1.00. The obtained results in the form of spectra distribution were accurately represented by simulations with the *3D model*. In the second stage, the iterative procedure for determining the polarization area of reinforcement in concrete was positively verified for additional test elements. Electrochemical parameters of the steel-concrete system were determined on the basis of the *3D model* with a simultaneous adjustment of the polarization area on the rebar. In this case, the expected matching degree of 1.00 was obtained for each tested system after more than ten iterations starting from matching the model spectra to the empirical spectra at the level of 0.31–0.93.

## 1. Introduction

The operational safety of large concrete structures can be provided by their administrators, who should have detailed knowledge of their technical conditions [1]. Facilities exposed to corrosive environmental factors in particular should be monitored on a regular basis with reference to corrosion risk [2,3,4,5,6,7,8]. Corrosion in reinforced concrete structures can be evaluated using three main types of testing. The first type includes tests on the protective properties of concrete against reinforcing steel. At first, concrete specimens are collected from the structure. Then, they are used for mapping pore solution and usually determining chlorides and pH [7,9,10]. Portable measurement kits for rapid and approximate in situ tests are often used [11]. The second group includes testing corrosion probability in a concrete reinforcement. As part of such tests, measurements of the stationary potential of reinforcing steel [12,13,14,15,16,17] and concrete cover resistivity are taken [18,19,20,21,22,23,24,25]. Taking into account the suitable criteria specified in standards, corrosion risk can only be predicted for this case. Electrochemical polarization tests are the third, and the most advanced, group of evaluation tests that provide the non-destructive testing of corrosion rate of reinforcing steel in concrete. Tests classified into this group usually employ the method of Linear Polarization Resistance (LPR) [6,14,17,26,27,28,29,30,31,32], Galvanostatic Pulse (GP) [33,34,35,36,37,38,39], and Electrochemical Impedance Spectroscopy (EIS) [38,39,40,41,42,43,44] to evaluate reinforced concrete. The analysed results and adequate criteria provide very accurate information on corrosion risk. However, the analysis of results obtained from polarization tests on reinforced concrete elements requires precise key identification during the analysis of the results for the test surface of reinforcement. This identification issue concerning the polarization surface has been a serious technical challenge for polarization testing of reinforced concrete since the beginning. There are a few published papers that demonstrate identification methods for this type of surface.

The most popular method consists of the application of twin counter electrodes placed in one head on the surface of the concrete cover over reinforcement. The auxiliary electrode is described as a guard ring that is used to confine distribution of polarization currents on the side surface of cylindrical reinforcement. The idea of guard ring has been repeatedly described and experimentally verified [35,45,46,47]. It was also a base for developing commercial devices to measure corrosion rate in concrete. Reliability and trueness of obtained results concerning the presence of some parallel or crossing rebars in concrete is disputable despite the popularity of this method.

The second method of identifying the polarization surface of reinforcement, which is also described in the available literature, is to perform a series of measurements, each time using a counter electrode of a different size. Finally, the distribution of outcomes of polarization resistance against the counter electrode surface can be analysed by extrapolating true values of polarization resistance for the projected unlimited polarization surface of the test electrode. The above testing method was described in the paper [17] as a concept and is classified as a very onerous method because measurements with different counter electrodes have to be repeatedly conducted.

The third, a laboratory and in situ verified method of identifying the polarization surface of reinforcement, is based on cutting a cylindrical core with part of the support rebar from the reinforced concrete structure. Then, a three-electrode system composed of an auxiliary electrode, a reference electrode, and a working electrode, which is a part of the cut rebar from the core, is arranged on the cylindrical core under laboratory conditions. Because geometry of the cut rebar is known, its side surface is also defined. Additionally, this side surface can be verified after crushing the core in the testing machine. Key information for testing the polarization surface of reinforcement is automatically acquired during the EIS measurements on such a core. Additionally, a possibility for simulating variable thermal and humidity conditions in the environment of the structure in a climatic and corrosion chamber provides a range of extreme values of the corrosion ratio instead of a single value obtained from traditional measurements. The above methodology for polarization tests on concrete cores was patented in 2012 and is also presented in the papers [30,48,49,50,51].

Another, fourth, method of identifying the polarization surface of reinforcement covered with concrete is to make a cylindrical cut in the concrete cover to a depth corresponding to its thickness prior to the EIS measurements. This circular cut should have a diameter equivalent to a cross-sectional diameter of the counter electrode, which is placed on the concrete surface through wet felt. The cut, which is recommended to be filled with liquid insulator, ensures relatively effective confinement of distribution of polarization currents over the side surface of steel reinforcement. The test surface of reinforcement can be estimated because the effects of the insulator are known. This methodology was patented in 2020, and its experimental verification is discussed in the papers [31,52,53]

In the search for non-destructive methods for identifying the polarization surface of reinforcing steel in concrete, this paper suggests a new methodology that requires only one EIS measurement with a single counter electrode and the analysis of results is enhanced with an original *3D model* of the *steel-concrete* system. The model, which is a base of the proposed methodology, has already been described and partially verified during tests concerning the impact of diameters [54] and the length of a rebar [55], the thickness of concrete cover [56], and the limited range of polarization [57] on the results of impedance measurements. The first stage of the discussed methodology is focused on the experimental evaluation of effects caused by the geometry of a rectangular counter electrode on the EIS tests for reinforced concrete, and the option of predicting (simulating) changes in shapes of impedance spectra using the original model. The second stage involves the experimental verification of this methodology on additional reinforced concrete elements. This method can iteratively determine electrochemical parameters and the key geometrical parameter—the polarization surface of reinforcement—using the assumptions of the developed *3D model*.

## 2. General Arrangements for the Original *3D Model* for Analysing the Steel-Concrete System by the EIS Method

**Figure 1:**
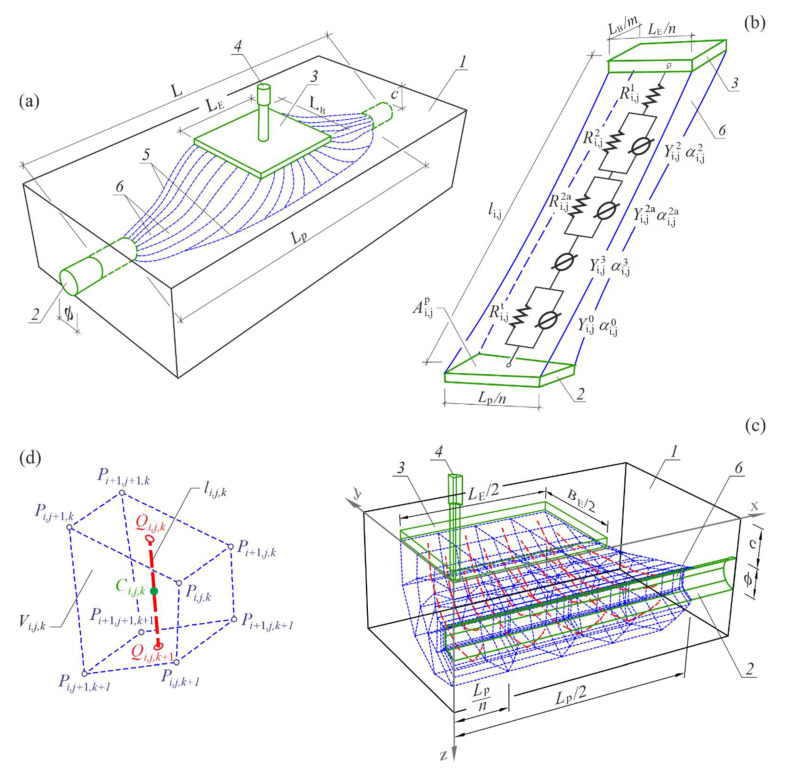
General arrangements for the *3D model* of the steel-concrete system (**a**) view of the modelled system with concrete volume 5 that was electrically conductive during the EIS measurements. This concrete volume included the polarized area of the rebar 2 on the section $L_{p}$, (**b**) the simplest specific case of the linear conductive path 6 between the counter electrode 3 and the working electrode 2, which was modelled with the basic equivalent electrical circuit, (**c**) a quadrant of the model sectioned off due to two planes of symmetry, (**d**) solid element of volume $V_{i,j,k}$ on the theoretical conductivity path with the pointed section of the theoretical line of current $l_{i,j,k}$.

The steel–concrete system composed of a rectangular concrete body 1 with a singular smooth rebar 2 of diameter *φ* and concrete cover of thickness c (Figure 1) was considered. A length of the interface between the rebar and concrete was L. The rectangular sheet $L_{E}\times B_{E}$, used as the counter electrode 3, was placed on the flat top surface of the concrete solid element through wet felt. The reference electrode 4 was inserted through a hole to the geometric centre of the counter electrode 3. 

The counter electrode 3, the reference electrode 4, and the working electrode 2 that was a steel rebar, formed a three-electrode system that was connected to the potentiostat.

For initially assumed range of polarization $\;L_{p}$ of the rebar and size of the counter electrode $L_{E}\times B_{E}$, the active concrete surface 5 during the flow of polarization currents is specified (Figure 1a). Conductive paths 6 are routed within the volume 5 between the counter electrode 3 and the rebar 2. The method of determining geometrical coordinates for electrically active surface of concrete 5 and geometry of conductive paths 6 is described in Appendix A. Identical singular electrical equivalent circuits were assigned to each theoretical conductive path (Figure 1b). A part of the diagram describing concrete contains the resistor $R_{i,j}^{1}\;$ representing the resistance of the liquid phase of concrete, the resistors $R_{i,j}^{2}$ and $R_{i,j}^{2a}$ describing the resistance of a double layer at the interface of the concrete–pore solution and constant phase elements *CPE*, described by the parameters $Y_{i,j}^{2}$, $\alpha_{i,j}^{2}$, $Y_{i,j}^{2a}$, $\alpha_{i,j}^{2a}$, which represent the pseudo capacitance of the double layer. On the other hand, a part of the diagram describing reinforcing steel contains the constant phase element *CPE* ($Y_{i,j}^{3}$, $\alpha_{i,j}^{3}$), which represents the transition zone between concrete and steel, and resistance of charge transfer $R_{i,j}^{t}$ through the pore solution-metal interface, which is connected in parallel with the pseudo capacitance of the double layer at the interface of pore solution–metal, described by the parameters $Y_{i,j}^{0}$, $\alpha_{i,j}^{0}$ of the constant phase element *CPE*. 

Definitions of local coefficients of concrete and steel geometry were introduced to map the irregular geometry of the flow area of polarization currents between the counter electrode 3 and the working electrode 2 in the *3D model*. The local coefficient for concrete geometry $\gamma_{i,j}^{c}$ on the conductivity path ij is determined from the following expression (1), according to the paper [54].

(1) $$\gamma_{i,j}^{c}=\sum_{k=1}^{p}\frac{l_{i,j,k}}{S_{i,j,k}}$$

where $l_{i,j,k}$ is a length of the current line in concrete element that forms a path of electrical conductivity (Figure 1b), given by the following Formula (2):

(2) $$l_{i,j,k}=\sqrt{{\left(x_{i,j,k}^{Q}-x_{i,j,k-1}^{Q}\right)}^{2}+{\left(y_{i,j,k}^{Q}-y_{i,j,k-1}^{Q}\right)}^{2}+{\left(z_{i,j,k}^{Q}-z_{i,j,k-1}^{Q}\right)}^{2}}$$

and $S_{i,j,k}$ is an arranged flow area of current in concrete solid element given by the expression (3):

(3) $$S_{i,j,k}=\psi_{i,j,k}\frac{V_{i,j,k}}{l_{i,j,k}},\;k=1,2,\ldots ,p$$

volume of the solid element $V_{i,j,k}$ in Equation (3) is defined as a triple integral (4):

(4) $$\begin{array}{c} V_{i,j,k}={∭}_{\Omega }f\left(x,y,z\right)\;dx\;dy\;dz,\\ i=1,2,\ldots ,\frac{n}{2},\;j=1,2,\ldots ,\frac{m}{2},\;k=1,2,\ldots ,p \end{array}$$

of a function of three variables $f\left(x,y,z\right)$, continuous and specified for each point of the analysed area of the solid body Ω. The parameters *n*, *m* and *p* mean a number of elements of the model structure towards *x*, *y*, and *z* axes, respectively. The approximated value of the integral (4) is determined using the algorithms of the Gaussian method for estimating the volume for a triple integral [58]. The solid body Ω restrained by six walls was defined with eight vertices (Figure 1d), whose coordinates were given by Formulas (A5)–(A8) presented in Appendix A. The coefficient $\psi_{i,j,k}$ in the Formula (3) is determined from the following relationship

(5) $$\psi_{i,j,k}=\frac{V_{i,j,k}^{w}}{V_{i,j,k}}=\frac{m_{i,j,k}^{w}}{m_{i,j,k}^{c}}\;\frac{\rho^{c}}{1}=w_{i,j,k\;}\rho^{c},$$

where $V_{i,j,k}^{w}$ is water volume in the total volume $V_{i,j,k}$ of the solid element. The volume of water $V_{i,j,k}^{w}$ was expressed as the quotient of water mass $m_{i,j,k}^{w}$ and its bulk density $\rho_{i,j,k}^{w}=$ 1 g/cm^3^, whereas the volume of the solid element was expressed as the quotient of the dry concrete mass $m_{i,j,k}^{c}$ and its bulk density $\rho_{i,j,k}^{c}=\rho^{c}=const$. The final version of the coefficient $\psi_{i,j,k}$ (5), which underwent elementary transformations, was simplified to the product of moisture content $w_{i,j,k}$ in the solid element of volume $V_{i,j,k}$ and bulk density $\rho^{c}$.

**Figure A1:**
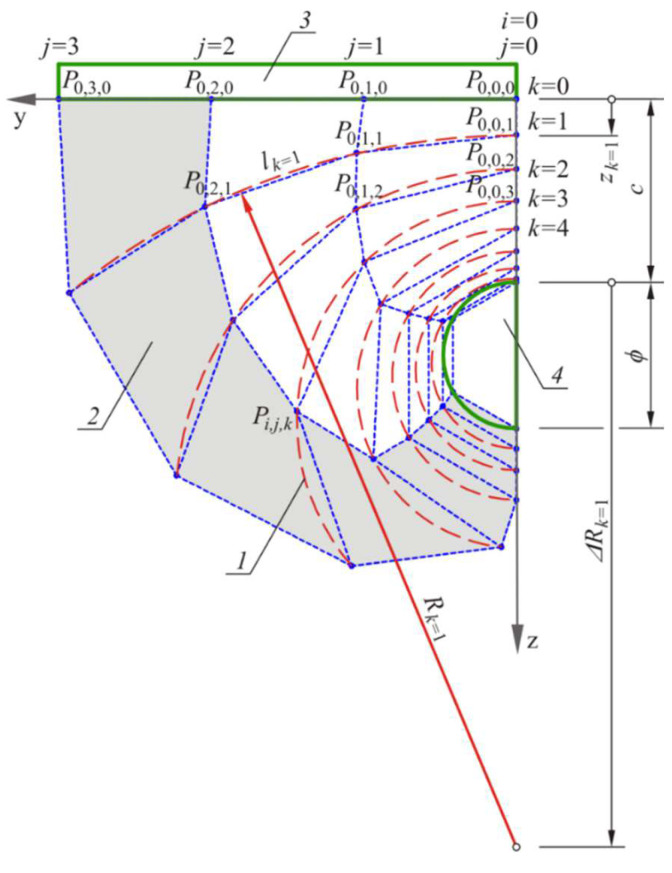
Criteria for determining guide parameters 1 for theoretical conductive paths 2 running between the counter electrode 3 and the rebar 4.

The local coefficient for steel geometry $\gamma_{i,j}^{s}$ is determined from the following expression (6) according to the paper [54]

(6) $$\begin{array}{c} \gamma_{i,j}^{s}=\frac{1}{A_{i,j}^{p}}\cdot \frac{l_{i,j}}{l_{mid}}=\frac{1}{\iint_{D}f\left(x,y,z\right)\;dD}\cdot \frac{\sum_{k=1}^{p}l_{i,j,k}}{\frac{\sum_{i=1}^{\frac{n}{2}}\sum_{j=1}^{\frac{m}{2}}l_{i,j}}{\frac{n}{2}\cdot \frac{m}{2}}},\\ i=1,2,\ldots ,\frac{n}{2},\;j=1,2,\ldots ,\frac{m}{2}, \end{array}$$

where $A_{i,j}^{p}$ is the elementary polarized area of the rebar at the end of the theoretical path of electrical (Figure 1b). The surface area $A_{i,j}^{p}$ was defined as the double integral of a function $f\left(x,y,z\right)$ of three variables, continuous and bounded within a rectangular area *D*. The approximated value of that integral was determined using the algorithms of the Gaussian method for estimating the volume for a double integral [58]. The flat quadrangle *D* with a surface area of $A_{i,j}^{p}$ was determined by four points $P_{i-1,j-1,k=p}$, $P_{i-1,j,k=p}$, $P_{i,j-1,k=p}$, $P_{i,j,k=p}$ located within an envelope of the rebar cross-section (cf. Figure A1 in Appendix A). Parameters $l_{i,j}$ and $l_{mid}$ from the Formula (6) are a length of the current line ij and an average length of all current lines on theoretical paths of electrical conductivity within the whole electrically-active volume of concrete 5.

Finally, the defined local coefficients of concrete $\gamma_{i,j}^{c}$ and steel $\gamma_{i,j}^{s}$ were used to form expressions (7) for global coefficients for concrete $\gamma^{c}$ and steel $\gamma^{s}$ [54]


(7) $$\gamma^{c}=\frac{1}{\sum_{i=1}^{\frac{n}{2}}\sum_{j=1}^{\frac{m}{2}}\frac{4}{\gamma_{i,j}^{c}}}\;\;\;\;\gamma^{s}=\frac{1}{\sum_{i=1}^{\frac{n}{2}}\sum_{j=1}^{\frac{m}{2}}\frac{4}{\gamma_{i,j}^{s}}}$$

They comprehensively describe the flow area of polarization currents between the counter electrode 3 and the working electrode 2 with reference to geometry of that area. The representative form of the expressions (7) resulted from two planes of symmetry included in the *3D model*. The total equivalent impedance $Z_{3D}$ of the tested system of steel–concrete (Figure 1) is given by the Formula (8):

(8) $$Z_{3D}=\frac{1}{\sum_{i=1}^{\frac{n}{2}}\sum_{j=1}^{\frac{m}{2}}\left(\frac{4}{Z_{ij}^{s}+Z_{ij}^{c}}\right)},$$

where:

(9) $$\begin{array}{c} Z_{ij}^{s}=\frac{R^{t}\frac{\gamma_{i,j}^{s}}{\gamma^{s}}}{1+R^{t}\frac{\gamma_{i,j}^{s}}{\gamma^{s}}Y^{0}\frac{\gamma^{s}}{\gamma_{i,j}^{s}}\omega^{\alpha^{0}}\left[\cos\left(\alpha^{0}\frac{\pi }{2}\right)+j\sin\left(\alpha^{0}\frac{\pi }{2}\right)\right]}+\\ +\frac{1}{Y^{3}\frac{\gamma^{s}}{\gamma_{i,j}^{s}}\omega^{\alpha^{3}}\left[\cos\left(\alpha^{3}\frac{\pi }{2}\right)+j\sin\left(\alpha^{3}\frac{\pi }{2}\right)\right]} \end{array}$$

(10) $$\begin{array}{c} Z_{ij}^{c}=R^{1}+\frac{R^{2}\frac{\gamma_{i,j}^{c}}{\gamma^{c}}}{1+R^{2}\frac{\gamma_{i,j}^{c}}{\gamma^{c}}Y^{2}\frac{\gamma^{c}}{\gamma_{i,j}^{c}}\omega^{\alpha^{2}}\left[\cos\left(\alpha^{2}\frac{\pi }{2}\right)+j\sin\left(\alpha^{2}\frac{\pi }{2}\right)\right]}+\\ \;\;\;\;\;\;\;\;\;\;+\frac{R^{2a}\frac{\gamma_{i,j}^{c}}{\gamma^{c}}}{1+R^{2a}\frac{\gamma_{i,j}^{c}}{\gamma^{c}}Y^{2a}\frac{\gamma^{c}}{\gamma_{i,j}^{c}}\omega^{\alpha^{2a}}\left[\cos\left(\alpha^{2a}\frac{\pi }{2}\right)+j\sin\left(\alpha^{2a}\frac{\pi }{2}\right)\right]} \end{array}$$

## 3. Studies on the Impact of Counter Electrode Geometry on Shapes of Impedance Spectra

Experimental tests related to the computational analysis with the *3D model* were divided into two stages. At first, assumptions made for the *3D model* were empirically verified with reference to the impact of the rectangular geometry of the counter electrode on shapes of impedance spectra.

### 3.1. Materials

Test elements were prepared from two concrete mixes, *S1* and *S2*. Concrete mix *S1* with the ratio of w/c = 0.43 was prepared from 489 kg/m^3^ of Portland cement 32.5R, 501 kg/m^3^ of fine aggregate with fraction to 2 mm, 1168 kg/m^3^ of coarse aggregate with fraction of 2–8 mm, and 212 L/m^3^ of water. Another mixture *S2* differed from the mixture *S1* only in the presence of an additive in the form of 3% NaCl dissolved in batched water. Smooth rebars with a diameter φ12 mm and φ16 mm, which were made from stainless steel of grade S235JR, were used as reinforcement in concrete elements. 

**Figure 2:**
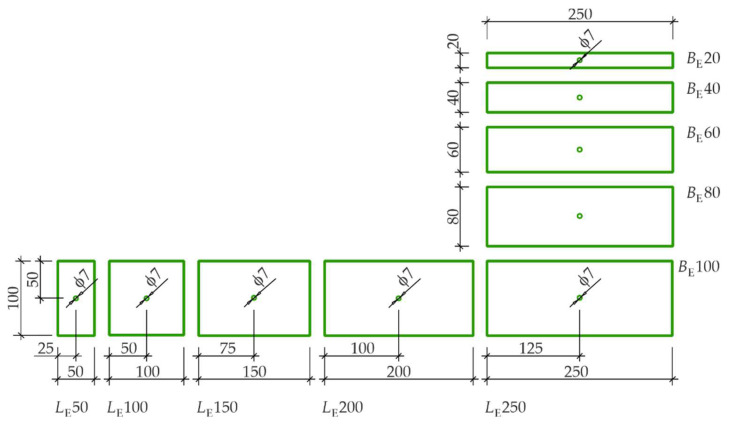
Stainless-steel counter electrodes used in the EIS tests on the impact of the auxiliary electrode geometry on the obtained shapes of impedance spectra.

The three-electrode system for the EIS measurements on reinforced concrete elements was prepared from a set of rectangular counter electrodes cut from a stainless steel sheet of grade X5CrNi18-9 (Figure 2). The counter electrodes formed a series of the fixed width $B_{E}$ = 100 mm and variable length $L_{E}$ = 50–250 mm, or the fixed length $L_{E}\;$= 250 mm and variable width $B_{E}$ = 20–100 mm. Besides the working electrode (the rebar) and the counter electrode, another Cl^–^/AgCl,Ag electrode was used as the reference electrode.

### 3.2. Measurement Arrangements and Their Model-Based Representation

**Figure 3:**
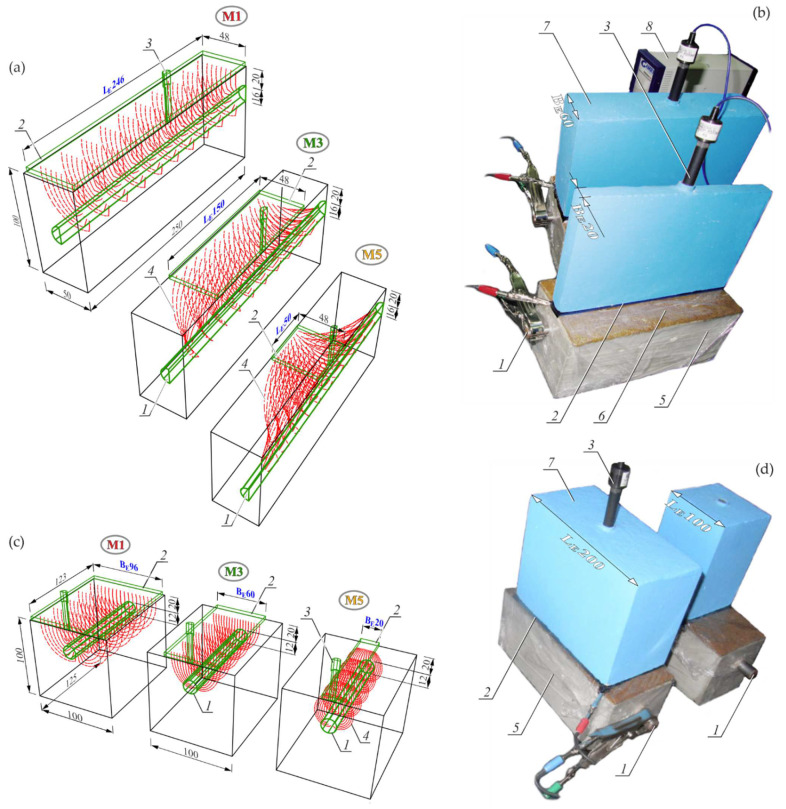
The three-electrode arrangements for testing the impact of the rectangular counter electrode geometry on shapes of impedance spectra of rebar in concrete: (**a**) *3D models* of the system with a variable length $L_{E}$ of the counter electrode, (**b**) the test stand with counter electrodes of variable widths, (**c**) *3D models* of the system with a variable width $B_{E}$ of the counter electrode, (**d**) the test stand with counter electrodes of variable lengths; 1—working electrode, 2—counter electrode, 3—reference electrode, 4—theoretical lines of current, 5—film, 6—felt, 7—ballast, 8—potentiostat.

The photo of a test stand for performing impedance tests on the impact of the rectangular counter electrode width on the shape of impedance spectra of steel in concrete is shown in Figure 3b, while the impact of the counter electrode length is presented in Figure 3d.

The impact of width $B_{E}$ was tested on two geometrically identical rectangular tests elements with dimensions of 250 × 100 × 100 mm, reinforced with single rebars with a diameter φ12 mm. The impact of the counter electrode length $L_{E}$ was also tested on two elements that were reinforced with rebars with φ16 mm. Two elements with rebars, φ12 mm and φ16 mm, were prepared from the concrete of series *S1*, which is characterized by high protective properties against steel. Two other elements were also made from concrete of series *S2* (with chloride additives) with reduced protective properties.

In accordance with the assumptions described in point 2, the test stand shown in Figure 3b was mapped with the model three-electrode systems presented in Figure 3a, whereas the test stand from Figure 3d was mapped with the model arrangements illustrated in Figure 3c. 

The working electrode in each case was the rebar 1 in concrete element, and the counter electrode 2 and the reference electrode 3, which were placed on the top surface of concrete through wet felt 6 to provide electric contact. Moisture content in concrete cover was measured with the dielectric method prior to each impedance measurement. The obtained results and the methodology for converting dielectric moisture into mass moisture are presented in Appendix B. Each of the test elements were protected with film *5* on each side to minimize the effect of a gradual change in moisture content in concrete cover.

Three characteristic models (*M1_L_*, *M3_L_* and *M5_L_*), which were selected from analysed five models presented in Figure 3a, differed mainly in a length $L_{E}$ of rectangular counter electrodes; that is, the size parallel to the rebar axis. For readability purposes, Figure 3a shows only half of the test element after the plane cut-off in the direction of the rebar axis. The result of a variable length of counter electrodes was a clearly different shape of the distribution of theoretical current lines *4* between the counter electrode 2 and the working electrode 1. Hence, the flow of alternating current through concrete during the impedance measurements was maximal at the longest counter electrode, and minimal at the shortest one. Similarly, Figure 3c presents the structural visualisation of three selected models (*M1_B_*, *M3_B_* and *M5_B_*) of the three-electrode system, representing the test stand from Figure 3d. For readability purposes, Figure 3c presents only half of the test element after the plane cut-off perpendicular to the axis of the rebar at its mid-length. The basic element that differentiated particular models was a length of the counter electrode 2, which had a very serious impact on the electrically active area of concrete. The visible shape of the current flow zone through concrete was closely related to the *3D model* assumptions describe in point 2 and to empirical equations given in Appendix A.

Table 1 presents geometric parameters of the models *M1_B_* and *M5_B_* and the moisture content of concrete, which were used in further computational analysis. The superscript *S1* at the symbol *M1_B_* in the table heading means that the spectrum *M1_B_* of the rebar φ12 mm with cover *c* = 20 mm, which was obtained at the counter electrode width $B_{E}$ = 96 mm, was the initial spectrum in the test element of series *S1*. Similarly, the superscript *S2* at the symbol *M_2B_* means that the spectrum *M2_B_* obtained for a narrower counter electrode with a width $B_{E}$ = 80 mm, was the initial spectrum in the test element of series *S2*. The term initial spectrum means any selected model spectrum, for which all geometric parameters are known, that is the base to generate other four model spectra according to the *3D model*, for which shapes are predicted at changing sizes of the counter electrode. The first model spectra *M1_B_* and *M2_B_* were obtained on the basis of the analysis of experimental spectra *P1_B_* and *P2_B_*.

In the context of testing the impact of the counter electrode length $L_{E}$, the geometric parameters of similar models *M1_L_* … *M5_L_* and moisture content in concrete, which were taken for the further computational analysis, are shown in Table 2. In this case, the symbol *M1_L_^S1^* means the spectrum selected as the first one in the element of series *S1* was the spectrum *M1_L_* obtained at the longest counter electrode $L_{E}$ = 246 mm, whereas the symbol *M4_L_^S2^* informs that the spectrum *M4_L_* obtained at $L_{E}$ = 100 mm was taken as the initial spectrum of the test element in series *S2*.

### 3.3. Comparative Assessment of Obtained Impedance Spectra

**Figure 4:**
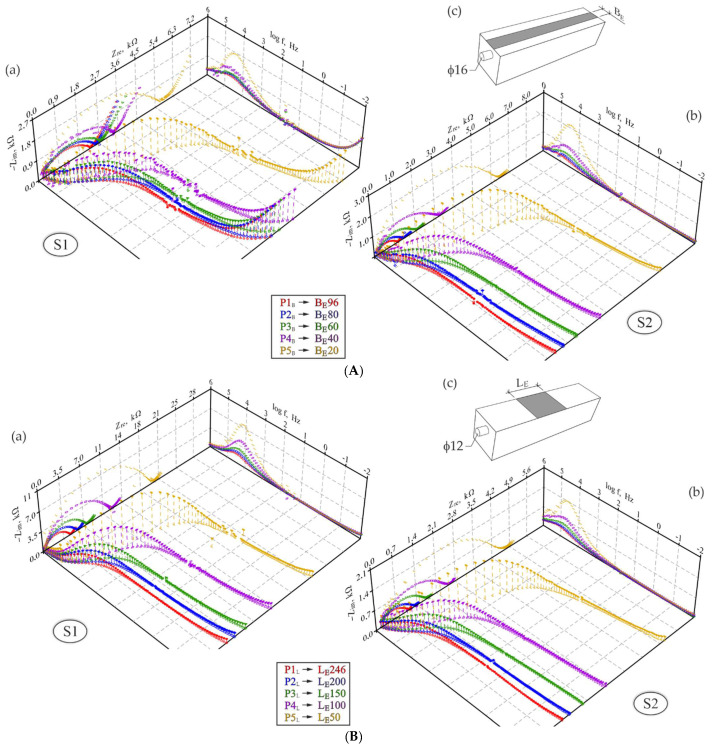
(**A**). Spatial diagrams (as a function of real impedance $Z_{re}$, imaginary impedance $Z_{im}$ and measurement frequencies f) representing distribution of impedance spectra obtained for: (a) and (b) rebars φ12 mm at a changing width $B_{E}$ of the counter electrode, in test elements of series *S1* and *S2*, respectively, (c) schematic drawing of the test element with a changing width $B_{E}$. (**B**). Spatial diagrams (as a function of real impedance $Z_{re}$, imaginary impedance $Z_{im}$ and measurement frequencies f) representing distribution of impedance spectra obtained for: (a) and (b) rebars φ16 mm at a changing length $L_{E}$ of the counter electrode in test elements of series *S1* and *S2*, respectively; (c) schematic drawing of the test element with a changing length $L_{E}$.

**Figure 5:**
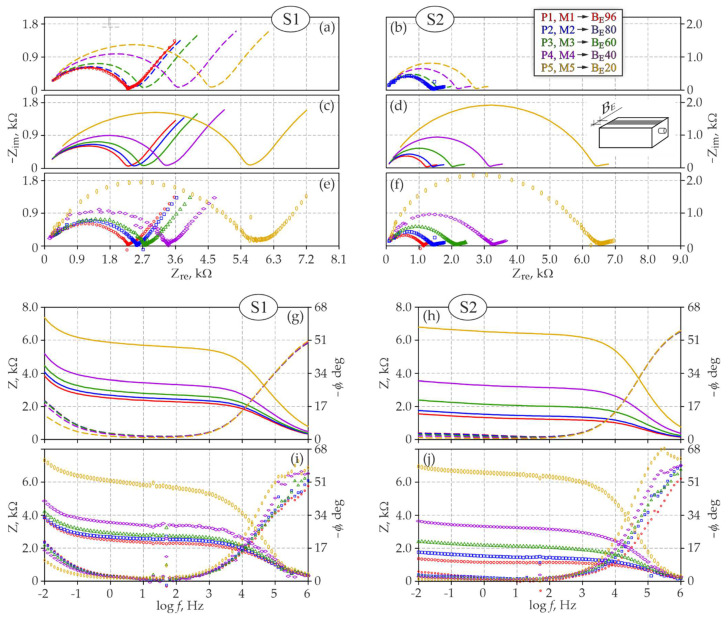
Comparison of test and simulation results concerning the impact of the counter electrode width $B_{E}$ on shapes of impedance spectra of steel in concrete of series *S1* and *S2*: (**a**,**b**) model spectra obtained on the Nyquist plot from initial spectra based on the *3D model*, (**c**,**d**) adjusted model spectra on the Nyquist plot that included moisture content in concrete, (**e**,**f**) experimental spectra on the Nyquist plot, (**g**,**h**) model spectra on the Bode plot with an included effect of moisture content in concrete, (**i**,**j**) experimental spectra on the Bode plot.

**Figure 6:**
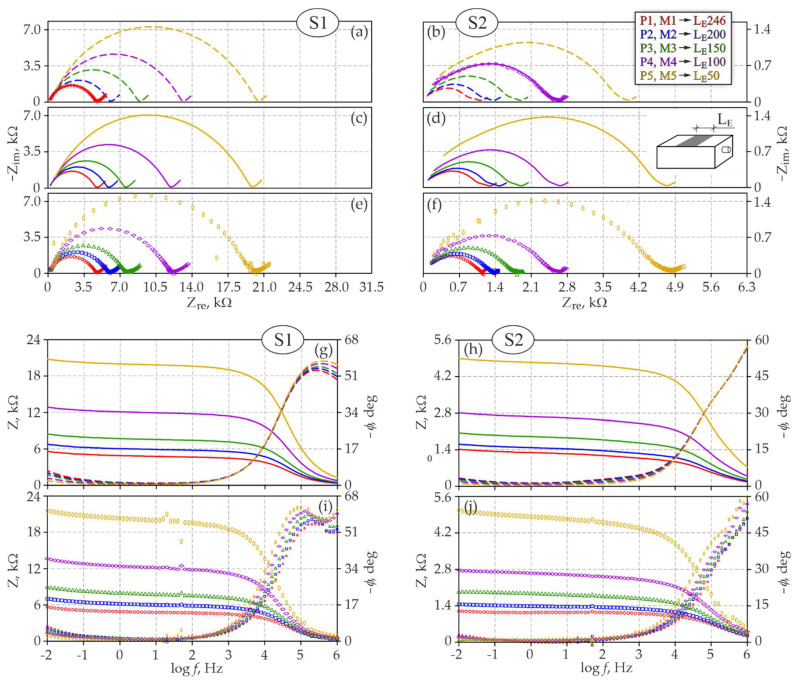
Comparison of model simulations and test results concerning the impact of the counter electrode length $L_{E}$ on the shapes of impedance spectra of steel in *S1* and *S2* concrete: (**a**,**b**) model spectra obtained on the Nyquist plot from initial spectra based on the *3D model*, (**c**,**d**) adjusted model spectra on the Nyquist plot that included moisture content in concrete, (**e**,**f**) experimental spectra on the Nyquist plot, (**g**,**h**) model spectra on the Bode plot with an included effect of moisture content in concrete, (**i**,**j**) experimental spectra on the Bode plot.

Figure 4A illustrates the perspective geometry of the distribution of impedance spectra obtained for steel rebars φ12 mm with concrete cover *c* = 20 mm at a changing width $B_{E}$ of the counter electrode in test elements of series *S1* and *S2*. Analogous plots for rebars φ16 mm at a changing length $L_{E}$ of the counter electrode are shown in Figure 4B. The same measurement results are presented in a traditional way on the Nyquist and Bode plots (Figure 5e,f,i,j and Figure 6e,f,i,j).

The comparative assessment of both groups of spectra concerning a changing width $B_{E}$ of the counter electrode (Figure 4A) clearly indicated similar impedance values measured for all steel–concrete systems. For the test elements of concrete of series *S1*, the impedance modulus for reinforcing steel was within a range of 3.9–7.4 kΩ (Figure 5i), while in concrete of series *S2*, the modulus range was slightly boarder 1.3–6.9 kΩ (Figure 5j). Contrary to the impedance moduli, phase shifts at the low-frequency ranges typical for steel were nearly four times greater for series *S1* than series *S2*. Moreover, phase shifts of elements of series S1 were becoming considerably larger at decreasing frequencies, which was opposite to the nearly steady path within the same range of frequencies for elements of series *S2*. These noticeable differences in phase shift distributions could indicate corrosion of reinforcement in concrete of series *S2* and steel passivation in concrete of series *S1*. The analysis of shapes of impedance spectra at the Nyquist plots confirms conclusions from the observations of the phase shift curve. Low-frequency arcs in concrete of series *S1* had a significantly wider slope angle and were much longer (Figure 5e) than very flat and short arcs in diagrams representing series *S2* (Figure 5f).

Apart from the above characteristics related to electrochemical properties of steel and concrete, particularly at the Nyquist plots (Figure 5e,f), also some characteristics related to a different geometry of tested steel-concrete-systems were observed. Distribution of points in test elements of series *S1* and *S2* was shifted towards the positive direction of the real impedance axis as the electrode width was reducing. Simultaneously very quickly changing representative diameter of high-frequency flat semi-circle at a minimal increase in a length of the low-frequency arc.

When comparing both groups of spectra (*S1* and *S2*) in terms of the impact of a changing length $L_{E}$ of the counter electrode on the impedance distributions, the first finding from analysing Figure 4B was over a five-fold difference in the scale of both components ($Z_{re}$, $Z_{im}$) of the impedance of the analysed steel–concrete system. The Bode plots in Figure 6 indicate that at low frequencies the maximal impedance modulus of the test element of series *S1* was within a range of 5.8–21.5 kΩ (Figure 6i), while for the element of series *S2* the modulus was within a much lower range of 1.2–5.2 kΩ (Figure 6j). Phase shifts on the same plots did not differ so significantly as the impedance moduli, but for the element of series *S1* slightly higher and more increasing values were observed at reducing frequencies. Both groups of spectra (*S1* and *S2*) on the Nyquist plots (Figure 6e,f) had very similar shapes, whereas the high-frequency semicircle was more flattened in concrete of series *S2*, the same referred to the low-frequency arc (Figure 6f). A narrow slope angle of this arc in reference to the axis of real impedance is often a symptom indicating the development of steel corrosion in concrete. The above qualitative analysis of spectra could indicate stronger stimulation of electrode processes on reinforcing steel in the test elements of series *S2* than *S1*.

Similarly, to the analysis of the impact of the counter electrode width $B_{E}$, a change in its length $L_{E}$ also led to very clear differences in spectra shapes. Because the impedance tests at different lengths of counter electrodes were conducted separately on two test elements, assuming that electrochemical state along the whole length of reinforcement in each concrete element was approximately the same, the clear differences in distributions of spectra were probably caused by the different geometry of individual three-electrode systems—cf. Figure 3a. Both groups of spectra on the Nyquist plots (Figure 6e,f) showed that at a decreasing length of the counter electrode, the representative diameter of the high-frequency semicircle, typical for concrete, considerably increased and, simultaneously, the low-frequency arc, typical for steel, was slightly elongated. Additionally, the relationship between the reduced length of the counter electrode and the increased representative diameter of the high-frequency circle was not linear.

### 3.4. Analysed Impact of the Counter Electrode Width $B_{E}$ on Impedance Shapes Based on the 3D Model

Test and simulation results based on the *3D model* and referring to the impact of the counter electrode width $B_{E}$ on shapes of impedance spectra of steel in concrete of series *S1* and *S2* are presented in the Nyquist and Bode plots (Figure 5). For ease of comparing model spectra and experimental spectra, the impedance distributions are illustrated on different plots.

**Figure 7:**
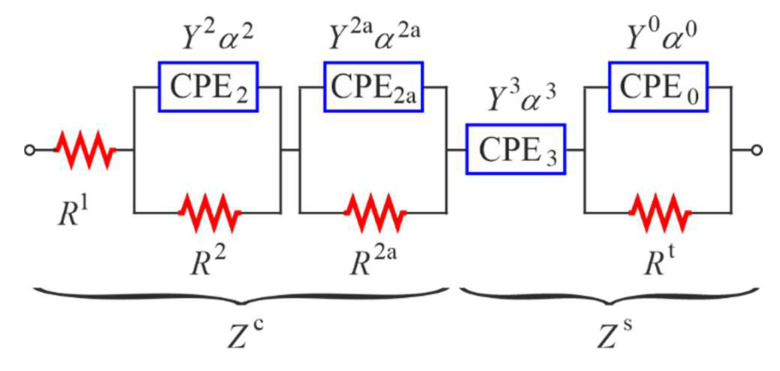
Electrical equivalent circuit used in the analysis of impedance spectra, in which: $R^{1}$—resistor representing resistance of liquid phase of concrete, $R^{2}$, $R^{2a}$—resistors describing resistance of a double layer at the interface of concrete–pore solution, $CPE_{2}$ and $CPE_{2a}$—constant phase elements described by the parameters $Y^{2}$, $Y^{2a}$, $\alpha^{2}$, $\alpha^{2a}$ which represents pseudo capacitance of the double layer, $CPE_{3}$—constant phase element with parameters $Y^{3}$, $\alpha^{3}$ which represents the transition zone between concrete and steel, $R^{t}$—the charge transfer resistance through the pore solution–metal interface, $CPE_{0}$—constant phase element with parameters $Y^{0}$, $\alpha^{0}$ which represent pseudo capacitance of the double layer at the interface of pore solution–metal; $Z^{c}$ and $Z^{s}$—groups of parameters describing concrete and steel, respectively.

At first, it was assumed that the polarization range in each model *M1_B_* … *M5_B_* was the same and equal to $L_{p}$ = 246 mm, which could be explained by the structure of the measuring systems—cf. Figure 3c,d. The spectrum *P1_B_* obtained for the reinforcement with the widest counter electrode $B_{E}$ = 96 mm was chosen as the initial spectrum for the test element of series *S1*. For the test element of series *S2*, the initial spectrum was the spectrum *P2_B_* obtained for reinforcing steel in concrete with the counter electrode with a width $B_{E}$ = 80 mm. Then, electrochemical parameters for steel and concrete were determined for the adopted electrical equivalent circuit (Figure 7) for both spectra *P1_B_* and *P2_B_*, using the method of iterative fitting. These values are shown in the second column of Table A1, Appendix C. The degree of fitting model spectra to the experimental ones was assessed with the coefficient q

(11) $$q=\sqrt{\frac{{𝒳}^{2}}{N-N_{p}}},\;{𝒳}^{2}=\sum_{i=1}^{N}\left[{\left(\frac{Z_{i}^{pom}-Z_{i}^{mod}}{\sigma_{i}}\right)}^{2}\right],$$

where: ${}^{2}$ is the objective function, N is the number of measuring frequencies for the impedance measurement, $N_{p}$ is the number of variables defined in the *3D model*, and parameters $Z_{i}^{pom}$ and $Z_{i}^{mod}$ mean values of impedance moduli for the *i*-th measuring frequency obtained from measurements and determined using the *3D model*. The parameter $\sigma_{i}$ is a standard deviation of the *i*-th measurement given by $\sigma_{i}=a\cdot Z_{i}^{pom}$, where a is the estimated measurement error expressed in percentage. The calculated coefficient q in concrete of series *S1* was 1.73, and in concrete of series *S2*—1.35 (Table A1).

In stage 2 of the analysis, the determined electrochemical parameters for both initial spectra *M1_B_* and *M2_B_* (Table A1) were put into Equations (8)–(10), which were used to obtain the predicted distributions of model spectra, illustrating the impact of a changing width of the counter electrode $B_{E}$. Dashed lines in Figure 5a indicate the curves of model spectra of reinforcement in concrete of series *S1* at the counter electrode widths *B_E_* = 80, 60, 40 and 20 mm, whereas in Figure 5b, dashed lines represent the spectra of concrete of series *S2* at the counter electrode widths *B_E_* = 96, 60, 40 and 20 mm. All model spectra in Figure 5a were obtained at average mass moisture in concrete equal to *w_mid_* = 4.7%, and the spectra in Figure 5b—at mass moisture *w_mid_* = 6.2%—cf. Table 1.

The solid line was used in the final stage of the analysis and shows in Figure 5c the distributions of model spectra *M2_B_*, *M3_B_*, *M4_B_* and *M5_B_* adjusted to spectra illustrated with dashed line in Figure 5a, while the solid line in Figure 5d presents the model spectra *M1_B_*, *M3_B_*, *M4_B_* and *M5_B_* adjusted to spectra presented in Figure 5b with the dashed line. Additionally, the adjusted model spectra are shown in the Bode plots—Figure 5g,h—the dashed line presents the adjusted model spectra but as a function of the logarithm of frequencies *f* and phase shift *φ*. By following the established methodology of the test analysis, the improved adjustment of model spectra to the theoretical spectra was obtained by iteratively choosing the theoretical mass moisture of concrete. Table 1 presents the theoretical moisture content values as an increase *Δw* in moisture content against mass moisture at which the initial spectra *M1_B_* and *M2_B_* were obtained. An increase in moisture content in concrete in test elements of series *S1* was observed within the range of +0.5%–−0.9%, whereas an increase within a wider range varying from +0.7% to 3.6% was observed for series *S2*. The determined values of theoretical moisture content, only with a certain approximation, represented the average mass moisture content in concrete, but, in accordance with the assumptions made for the *3D model*, these values also directly included the spatial inhomogeneity of the porous structure of concrete through the coefficient $\psi_{i,j,k}$, expressed with the Formula (5). The inhomogeneity of the concrete structure, mainly its non-uniform dampness, could be explained by different values of theoretical moisture content in one test element. The model simulations of current flow through concrete (Figure 3c) indicate that the concrete area, which was active during the impedance measurements and increased with the increasing width of the counter electrode, could have the average moisture content.

### 3.5. Analysed Impact of the Counter Electrode Length $L_{E}$ on Impedance Shapes, Based on the 3D Model

Simulation and test results based on the *3D model* are compared on the Nyquist and Bode plots (Figure 6) and refer to the impact of the counter electrode length $L_{E}$ on shapes of impedance spectra of steel in concrete of series *S1* and *S2*.

At the beginning of the analysis, the same polarization range *L_p_* = 246 mm was assumed for all models *M1_L_* and *M5_L_*. Contrary to the above discussed impact of the counter electrode width on the distribution of the impedance spectra, the assumption that the polarization could cover the whole reinforcement area at each, even the shortest counter electrode, was not so obvious. Therefore, additional impedance tests were performed on long concrete test elements of 1000 × 100 × 56 mm, reinforced with a single rebar φ16 mm (cf. Section 4.1) to alleviate any concerns. On the basis of the analysed additional verification tests (whose results were not presented in this paper), the very strong assumption was made that polarization currents in four times shorter test elements; that is, 250 mm long, would cover the whole length of rebar in concrete at each of the five lengths of the counter electrode $L_{E}$.

The spectrum *P1_L_* obtained for the longer counter electrode *L_E_* = 246 mm was regarded as the initial spectrum for the test element of series *S1*, while for the test element of series *S2,* the optimum spectrum for model simulations was the spectrum *P4_L_* obtained for the counter electrode length *L_E_* = 100 mm. Then, electrochemical parameters for concrete and steel were determined for the adopted electrical equivalent circuit (Figure 7) for both spectra *P1_L_* and *P4_L_*, using the method of iterative fitting. These values are shown in Table A2, Appendix C. Matching model spectra to the experimental ones using the coefficient q described by Equation (11), the value of 1.14 was obtained for the element of series *S1*, and q = 0.96 for the element of series *S2* (Table A2).

Similarly to the modelled effect of the counter electrode width, the 2nd stage of the analysis provided predicted distributions of model spectra that showed the effect of a decreasing length of the counter electrode after entering into Equations (8)–(10) electrochemical parameters determined from Table A2—Figure 6a,b.

Dashed lines in Figure 6a illustrate distributions of model spectra of the element of series *S1* obtained from the counter electrodes of *L_E_* = 200, 150, 100 and 50 mm in length, at the average mass moisture of concrete *w_mid_* = 4.8%. The spectra in Figure 6b show the spectra of the element of series *S2* corresponding to the following lengths: *L_E_* = 246, 200, 150 and 50 mm, obtained at the measured average moisture content in concrete *w_mid_* = 5.0%—cf. Table 2.

The solid line in Figure 6c presents the adjusted model spectra *M2_L_*, *M3_L_*, *M4_L_* and *M5_L_* of the test element of series *S1*, while spectra *M1_L_*, *M2_L_*, *M3_L_* and *M5_L_* in Figure 6d refer to the element of series *S2*. As in the previous analysis of the impact of the counter electrode width, the adjusted model spectra are additionally shown on the Bode plots—Figure 6g,h. Additionally, in this case, matching of model spectra to the theoretical ones was adjusted by the iterative method specifying the theoretical mass moisture of concrete, which is presented in Table 2 as an increase *Δw* in moisture content against mass moisture, at which the initial spectra *M1_L_* and *M4_L_* were obtained. An increase in moisture content in concrete element of series *S1* was observed within a relatively narrow range of +0.2–+0.9%, whereas moisture content in concrete of series *S2* was close to the absolute value and ranged from −0.1 to 0.9%. As in the case of analysing the impact of the counter electrode width, the non-uniform moisture content in concrete was the reason for varying theoretical values of average moisture content in concrete per the element volume. In relation to the simulated distribution of current lines shown in Figure 3a, changing length of the rectangular counter electrode had a very strong effect on the concrete area that was active while reinforcement polarization was measured. Therefore, each of the examined three-electrode systems with a different length of the counter electrode due to different zones of current flow through concrete, could be described by different average moisture content in concrete.

## 4. Iterative Method of Determining the Polarization Surface of Reinforcement in Concrete 

The experimental verification presented in Section 3 refers to the impact of the changing geometry of the rectangular counter electrode on the shapes of impedance spectra and lays the foundations for developing a new methodology for determining the polarized area of reinforcement. The described tests that employ the counter electrode complement previously published studies [54,55,56,57] that confirm a possible prediction and a *3D model*-based simulation of effects on the impedance shapes produced by the reinforcement diameter and length, concrete cover, and limited polarization range forced by an electrical insulator. 

### 4.1. Materials and Measuring System

For the purpose of showing the functionality of the elaborated iterative method of determining polarized area of reinforcement in concrete of series *S1*, an elongated test element with the dimensions of 1000 × 100 × 56 mm was prepared That element was reinforced with a bar φ16 mm, 1030 mm long, made of smooth steel of S235JR grade. A substantial length of the reinforced concrete element, in the context of the electrochemical tests, was to simulate measurements on real elements from concrete structures under laboratory conditions. Additionally, this element could prevent polarization of the whole rebar covered with concrete at the considerably small size of the counter electrode. Otherwise, the method functionality could not be effectively assessed.

**Figure 8:**
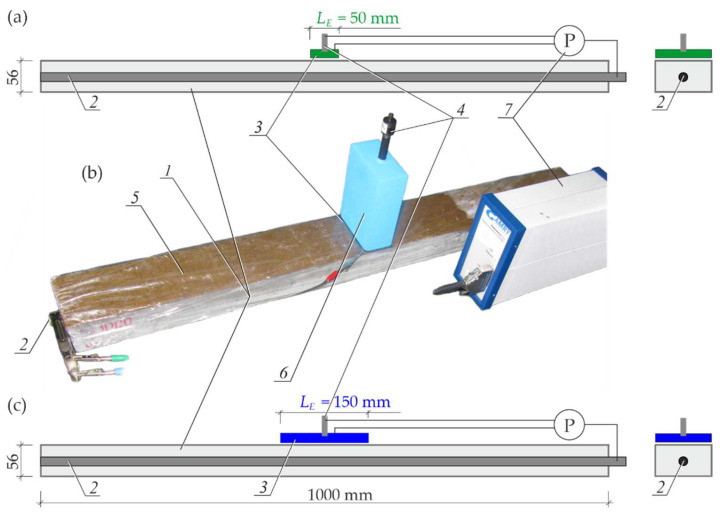
The test stand for verification of the iterative method for determining the polarized area of reinforcement during the EIS tests on reinforced concrete: (**a**) longitudinal and transverse sections of the system with a shorter auxiliary electrode, (**b**) a photo of the test stand, (**c**) longitudinal and transverse sections of the system with a longer auxiliary electrode; 1—concrete test element, 2—working electrode, 3—counter electrode, 4—reference electrode, 5—felt, 6—ballast, 7—potentiostat.

A view of the test stand for impedance measurements of long test elements 1 is presented in Figure 8. The three-electrode system contained the working electrode 2, the rebar, the auxiliary electrode 3, a rectangular stainless-steel sheet, and the reference electrode 4 and the silver chloride electrode. A wet felt 5, pressed down by concrete ballast 6, was put between the counter electrode 3 and the top part of the concrete to provide the satisfactory electrical contact. A vertical hole in the ballast was used as a guide for the reference electrode 4. The whole concrete element and the wet felt were protected with film to provide stable moisture conditions. Those three electrodes were connected to the potentiostat 7 *Gamry Reference 600*, which was used in the testing procedure for measuring impedance at a rage of frequencies of 1 mHz–1 MHz at the potential amplitude 20 mV in potentiostatic mode.

The EIS measurements were taken for comparative analysis of two counter electrodes with a length of $L_{E}$ = 50 mm and $L_{E}$ = 150 mm, respectively, and the same width $B_{E}$ = 100 mm equal to a width of the test element. Results from both measurements are shown as the distribution of points on the Nyquist plot and the Bode plots in Section 4.2.

### 4.2. Procedure for Determining Polarization Surface of Reinforcement in Concrete, Based on the 3D Model

**Figure 9:**
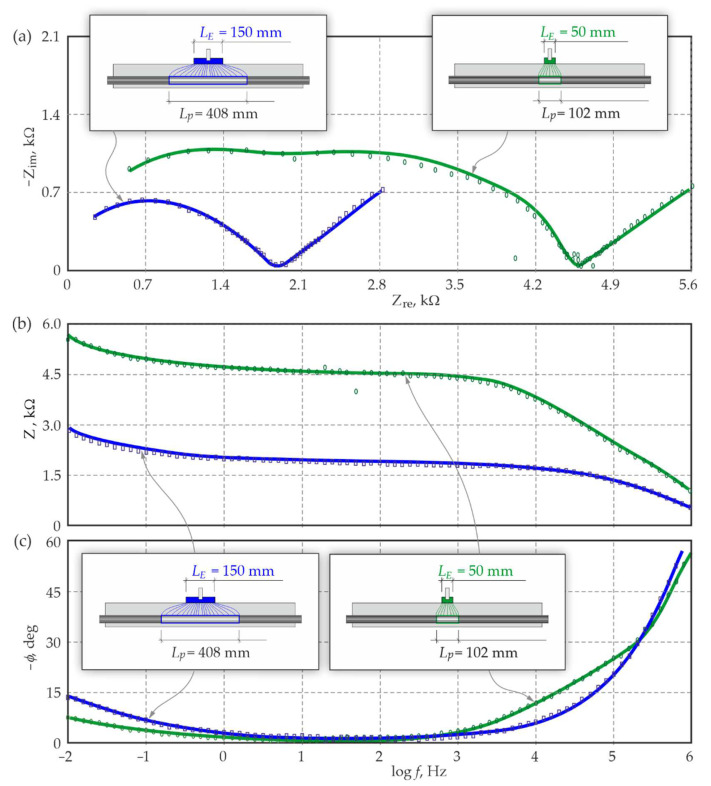
Comparative presentation of matching the model spectra (solid lines) to experimental spectra (points) for two lengths *L_E_* of counter electrodes, according to the original iterative method for determining the polarized area of reinforcement during the EIS tests on reinforced concrete: (**a**) the Nyquist plot, (**b**) the Bode *Z* (log *f*) plot, (**c**) the Bode *φ* (log *f*) plot.

Electrochemical parameters of the model were determined in the first stage of tests using the equivalent electrical circuit, as shown in Figure 7, separately for both experimental spectra (point distributions are presented in Figure 9). Values of these parameters are shown in Table A3, Appendix C.

In the second stage, these Formulas (1)–(7) were used to calculate local and global coefficients of concrete and steel geometry, for which lengths of polarization ranges $L_{p}$ corresponding to lengths of both verified counter electrodes $L_{E}$ were provisionally assumed. Then, after introducing the calculated geometry coefficient into the Formulas (9) and (10), the shapes of model spectra were generated on the Nyquist and Bode plots on the basis of the Formula (8). The model spectra with the colourful distinction for the shorter counter electrode $L_{E}\;$= 50 mm (green colour) and the longer one $L_{E}$ = 150 mm (blue colour), are presented by solid lines in Figure 9.

The coefficient q expressed by the Formula (11) was used to evaluate the matching degree of model spectra to the experimental. The obtained coefficient for the shorter counter electrode was q = 0.93, whereas for the longer one, the coefficient was q = 0.31. In accordance with the developed methodology, the expected value of the coefficient q indicating the optimum matching of the spectra was equal to 1, then the expected value of the coefficient q = 1.00 was determined in the third and final stage of the analysis at gradually increasing or decreasing length $L_{p}$, separately for both counter electrodes.

The selected values of the coefficient q determined by the iterative procedure for spectra matching are presented in Table 3, individually for each counter electrode. Finally, the polarization range of a steel rebar determined form the *3D model* for the shorter counter electrode $L_{E}\;$= 50 mm was $L_{p}\;$= 102 mm, and for the longer counter electrode, this range was $L_{E}$ = 150 mm—$L_{p}\;$= 408 mm.

Regarding the corrosion evaluation of reinforced concrete, density of corrosion current $i_{\mathrm{corr}}$ was the key information obtained from the EIS tests performed on reinforcement in concrete. Therefore, knowing the polarization range $L_{p}$ the polarized area was determined from the basic Formula $A_{p}=\pi \phi L_{p}$, and then, the Stern-Geary Equation [59,60] was used to calculate $i_{\mathrm{corr}}$.

(12) $$i_{\mathrm{corr}}=\frac{B}{R_{p}A_{p}}$$

where the polarization resistance $R_{p}$ approximately corresponds to the resistance of charge transfer $R_{t}$ [61]. The coefficient B present in the Formula (12) and dependent on Tafel constants for the anodic and cathodic reactions, was considered as equal to 52 mV [61,62,63,64], due to the preliminary assumed passivation state of steel in concrete.

Taking into account the above assumptions and substituting values of the charge transfer resistance $R_{t}$, taken from Table A3 (Appendix C), into the Formula (12), densities of corrosion current were calculated. The value for the shorter counter electrode was $i_{\mathrm{corr}}$ = 0.80 μA/cm^2^, and for the longer one was $i_{\mathrm{corr}}$ = 0.86 μA/cm^2^. As can be seen, the values $i_{\mathrm{corr}}$ calculated for different counter electrodes were very close because they referred to the same rebar in concrete. However, they were not the identical values because polarized areas of the same rebar differed for different counter electrodes and, locally, they could have different electrochemical conditions.

**Figure 10:**
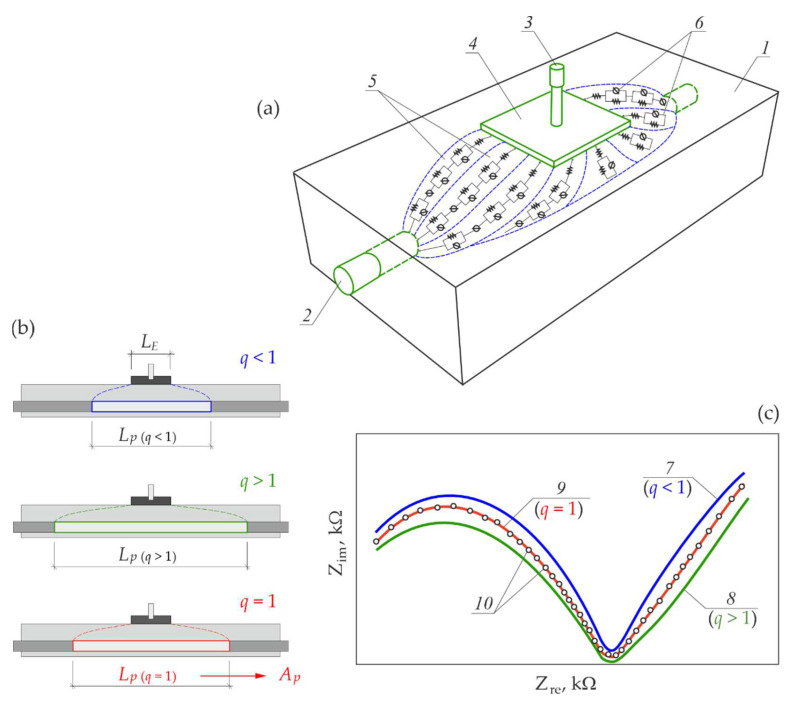
Schematic presentation of the iterative methodology for determining the polarized area of reinforcement in concrete using the *3D model*: (**a**) main assumptions for the *3D model* presented in a visual form: 1—concrete, 2—rebar, 3—reference electrode, 4—counter electrode, 5—theoretical paths of electrical conductivity of concrete, 6—basic electric equivalent circuits at each conductive path, (**b**) iteratively selected ranges $L_{p}$ of reinforcement polarization and corresponding factors q of matching degree of spectra, (**c**) shapes of model spectra 7, 8, 9 obtained in the complex plane according to the *3D model* and corresponding factors q of the empirical degree of spectrum matching 10; based on the range $L_{p}$ determined for q=1 calculated as the polarized area of reinforcement $A_{p}$.

To summarise the above iterative procedure for determining the polarized area of reinforcement during the measurements on reinforced concrete corrosions by the EIS method, the schematic presentation of key elements and stages of this methodology are shown in Figure 10.

## 5. Summary and Conclusions

- This paper supplements multi-thread experimental tests that verify the original *3D model*, which could include and separate at the analysis stage features of impedance spectra not related to electrochemical effects. The development of an iterative methodology for determining the polarized area of reinforcement in a single rebar in concrete highlighted the practical functionality of the model.
- A novelty of the experimentally verified *3D model* was to define the area of electrically conductive concrete within the developed three-electrode system (with a rebar as the working electrode) and route theoretical paths of current in that area. Basic equivalent electrical circuits, which were connected in parallel, were assigned to each current path, which was a curved block of concrete between the counter electrode and the working electrode. By introducing the geometric coefficient for concrete and steel into Formulas expressing the overall impedance of the system, electrochemical parameters of equivalent circuits were coupled with geometric parameters.
- Relying on empirical Formulas (Appendix A) to determine the electrically conductive area of concrete during the flow of polarization currents and on empirical relations to route theoretical conductive paths was the main drawback and significant limitation to the application of that *3D model*. The future transition into solutions related to the method of finite elements would lead to increased accuracy of modelling the *steel-concrete* systems; however, it will be a difficult task.
- Results of impedance tests described in this paper referred to the experimental verification of the final, not tested up to then, geometric parameter of the *3D model*; that is, the dimensions of the rectangular counter electrode. Other geometric parameters of the analysed *steel-concrete* system; that is, a diameter and length of the rebar, thickness of the cover, and polarization range, have already been positively verified and documented in the papers [54,55,56,57]. Matching degrees of model spectra to the experimental spectra obtained from the statistical analysis were q = 1.35–1.73 for the tests on the width effect $B_{E}$, and for the length effect $L_{E}$ of the counter electrode they were q = 0.96–1.14.
- The discussed impedance tests performed for different widths and lengths of the rectangular counter electrode indicated a strong relationship between the spectra shapes and variable geometry of the analysed steel-concrete system. The verified *3D model* represented tendencies for impedance shapes to change, which was observed during the EIS measurements. However, a slight adjustment of model spectra by a relevant selection of the theoretical moisture content in concrete had a significant impact on the matching degree.
- The tests employed a simplified method of measuring moisture content in concrete with a dielectric method and converting this parameter into mass moisture (Appendix B). If the precise identification of the spatial distribution of moisture content in an electrically conductive area of concrete could be technically possible during the polarization, it would minimize the observed discrepancies. The greatest discrepancies between the model and empirical spectra were found within a high-frequency range, with reference to the phase shift in the Bode plots—cf. Figure 5g–j and Figure 6g–j.
- The main objective of this paper was to present and experimentally verify the original iterative method for determining the polarized area of reinforcement in concrete. Based on the developed methodology, the initial matching degree of model spectra to the empirical spectra was q = 0.31–0.93, and after more than ten iterations in each, both test systems reached the expected value q = 1.00. This procedure, which is schematically presented in Figure 10, was based on the assumption made for the *3D model* and the relevant formulas. Hence, a description of this method required a complex presentation of the model (Section 2).
- The iterative method for determining the reinforcement polarization involved the use of a single counter electrode in the three-electrode system and the possible identification of the test area, including electrochemical parameters obtained from a single EIS measurement. Therefore, the experimental tests to verify the simulated impact of varying sizes of the counter electrodes on the spectra shapes, based on the *3D model*, was found to be important in this paper.
- It is difficult to compare this new method of determining polarization surface of reinforcement in concrete with other methods that include the analysed surface in measurements of the reinforcement corrosion rate, which were specified in the Introduction section, in a measurable way without additional tests. The proposed approach seems to be closest to a common solution based on measurements using a counter electrode with guard ring.
- Looking ahead to further developments of this model to associate it with the method of finite elements, there is the possibility of implementing the described algorithms into the software of testing devices that could be used to provide a more precise in situ evaluation of risk corrosion of reinforcement in concrete.

## Figures and Tables

**Table 1:** Geometric parameters of the *3D model* at a variable width $B_{E}$ of the counter electrode and average moisture content in concrete considered in the computational analysis of impedance spectra of steel in concrete of series *S1* and *S2*.

Parameters					*3D Model*		
			*M1_B_^S1^*	*M2_B_^S2^*	*M3_B_*	*M4_B_*	*M5_B_*
*S1* *S2*	*φ*,	mm	12				
	c,	mm	20				
	$B_{E}$,	mm	96	80	60	40	20
	$L_{E}$,	mm	246				
	L,	mm	250				
	$L_{p}$,	mm	246				
	$A_{p}$,	cm^2^	92.64				
	$\gamma^{s}$,	cm^−2^	0.008366	0.008828	0.009504	0.01016	0.01006
*S1*	$\gamma^{c}$,	cm^−1^	0.1805	0.1933	0.2141	0.2654	0.4552
	$w_{mid}$,	%	4.7	*Δw* = −0.1	*Δw* = 0	*Δw* = +0.5	*Δw* = −0.9
*S2*	$\gamma^{c}$,	cm^−1^	0.1244	0.1441	0.2064	0.3246	0.6639
	$w_{mid}$,	%	*Δw* = +0.7	6.2	*Δw* = −1.3	*Δw* = −1.9	*Δw* = −3.6

**Table 2:** Geometric parameters of the *3D model* at a variable length $L_{E}$ of the counter electrode and average moisture content in concrete considered in the computational analysis of impedance spectra of steel in concrete of series *S1* and *S2*.

Parameters					*3D Model*		
			*M1_L_^S1^*	*M2_L_*	*M3_L_*	*M4_L_^S2^*	*M5_L_*
*S1* *S2*	*φ*,	mm	16				
	c,	mm	20				
	$B_{E}$,	mm	96				
	$L_{E}$,	mm	246	200	150	100	50
	L,	mm	250				
	$L_{p}$,	mm	246				
	$A_{p}$,	cm^2^	123.53				
	$\gamma^{s}$,	cm^−2^	0.006419	0.006665	0.006903	0.006916	0.006806
*S1*	$\gamma^{c}$,	cm^−1^	0.1618	0.2032	0.2630	0.4233	0.7132
	$w_{mid}$,	%	4.8	*Δw* = +0.2	*Δw* = +0.9	*Δw* = +0.5	*Δw* = +0.2
*S2*	$\gamma^{c}$,	cm^−1^	0.1935	0.2286	0.3087	0.4584	0.8706
	$w_{mid}$,	%	*Δw* = −0.9	*Δw* = −0.5	*Δw* = −0.1	5.0	*Δw* = −0.9

**Table 3:** Comparison of values of the spectra matching factor q for statistical evaluation of iterative determination of the polarization range $L_{p}$ of reinforcement in concrete at the shorter counter electrode $L_{E}$ = 50 mm and the longer counter electrode $L_{E}$ = 150 mm.

$L_{E}=50\mathrm{mm}$		$L_{E}=150\mathrm{mm}$	
$L_{p}\;[\mathrm{mm}]$	q	$L_{p}\;[\mathrm{mm}]$	q
100	0.93	150	0.31
200	1.27	250	0.40
110	0.96	350	0.78
105	0.98	450	1.14
103	0.99	400	0.97
102	1.00	420	1.05
		410	1.01
		408	1.00

**Figure A2:**
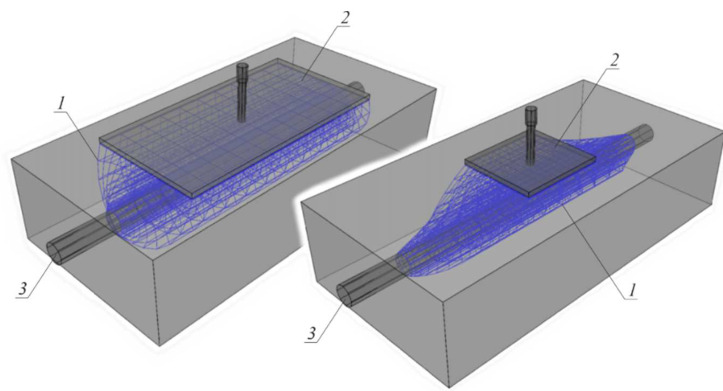
Comparison of the “flexible” grid of points representing electrically conductive area of concrete 1 under the alternating current polarization between the counter electrode 2 and the rebar 3; point grids shown in the figure were determined empirically from the Formulas (A1)–(A16).

**Figure A3:**
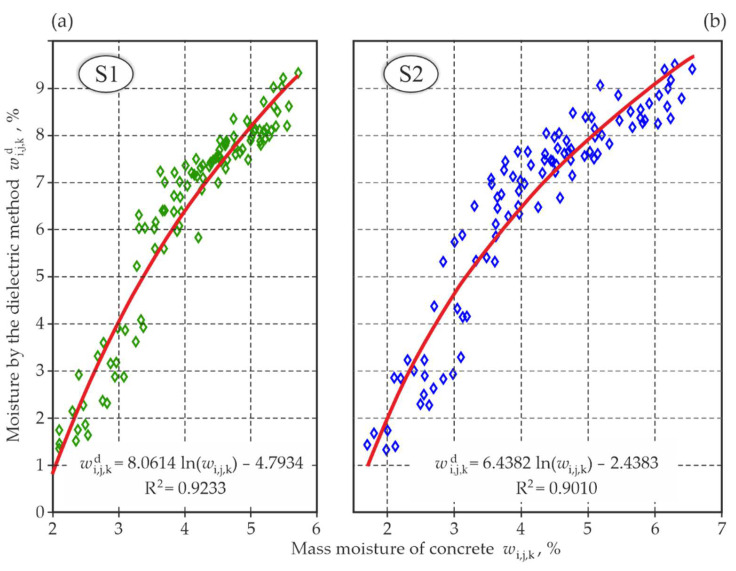
Results from correlation tests between the measurements of moisture content by the drying and weighing method and the dielectric method: (**a**) concrete with the best protective properties—series *S1*, (**b**) concrete with chlorides and reduced protective properties—series *S2*.

## Data Availability

The data presented in this study are available on request from the corresponding author.

## Appendix A

To determine the theoretical paths of electrical conductivity in the conductive area of concrete under alternating current polarization, the model structure (Figure 1a,c) was divided in the direction of *x* axis into *n* elements, in the direction of *y* axis into *m* elements, and in the direction of *z* axis into *p* elements (*n*, *m*—even numbers). As a consequence of that division, both the counter electrode 3 and the working electrode 2 consisted of the same number of *n* × *m* surface elements (Figure 1b). They are named surface elements because their thickness can be neglected while describing the observed physico-chemical process. However, concrete between the counter electrode 3 and the working electrode 2, serving as the electrically conducting medium, was divided into irregular octagonal solid elements in a number of *n* × *m* × *p* (Figure 1d). On the basis of the above assumptions, the number of theoretical conductive paths in concrete was *n* × *m*, and each path consisted of the same *p* number of connected segments made of connected solid elements (Figure 1c).

Due to strongly variable geometry of the flat counter electrode and the working electrode oval in cross-section, the analysed area of flow of the polarization currents was characterized by great irregularities in shapes. The assumed division of the model into the surface and solid elements defined the geometrical relationships to determine the coordinates creating the spatial grid of the model nodes (Figure 1d). For that purpose, the auxiliary elements in the form of current path guides were introduced—Figure A1. Each guide 1 was an arc of radius $R_{k}$ and length $l_{k}$, and the centre located at a distance $\Delta R_{k}$ (measured towards the positive direction of the *z* axis) from the point in the perimeter of the rebar cross-section, which was the closest to the counter electrode.

All of those three fundamental parameters of the guides ($R_{k}$, $l_{k}$, $\Delta R_{k}$) and the auxiliary parameter $z_{k}$—the shortest distance between the arc guide and the *x*-*y* plane, can be derived from the following Relationships (A1)–(A4) after introducing simple conversions:

(A1) $${\left(1-\frac{\frac{\varphi }{2}}{R_{k}}\right)}^{2}=1-sin\left(\frac{k}{p}\cdot \frac{\pi }{2}\right)\to R_{k},\;k=0,1,\ldots ,p$$

(A2) $$\Delta R_{k}=R_{k}-c{\left(1-\frac{\frac{\varphi }{2}}{R_{k}}\right)}^{2},\;k=1,2,\ldots ,p$$

(A3) $$l_{k}=B_{E}+\left[\left(B_{p}-B_{E}\right)\frac{k}{p}\right]+\left(\frac{c}{\varphi }\frac{\left|B_{p}-B_{E}\right|}{2}\right)\cdot \sin\left(\frac{k}{p}\pi \right)\leq 2\pi R_{k},k=1,2,\ldots ,p$$

(A4) $$z_{k}=c\cdot sin\left(\frac{k}{p}\cdot \frac{\pi }{2}\right),\;k=0,1,\ldots ,p$$

It can be observed that for k = 0 according to (A1) $R_{k}$ → ∞, and according to (A4) $z_{k}$ = 0, which means that the guide overlapped with the counter electrode on the outer surface of concrete. While for *k* = *p* according to (A1) $R_{k}$ = *φ*/2, that is, the radius of the guard arc corresponded to the radius of the rebar cross-section, and according to (A2) *ΔR_k_* = *φ*/2, which means that a centre of the guide arc was in the middle of the rebar cross-section. On the other hand, according to (A3) $l_{k}=B_{p}=\pi \varphi$, which means the guard arc changed into a circle with a radius equal to the radius of the rebar- cross-section, and according to (A4) $z_{k}=c$, that is, the guard was at a maximum distance of the cover thickness from the outer surface of concrete.

The Relationships (A1)–(A4) were used to develop the Formulas for determining the coordinates of the spatial grid of the model nodes $P_{i,j,k}$—Figure A1 and Figure 1d

(A5) $$P_{i,j,k}\left(x_{i,j,k}^{P},y_{i,j,k}^{P},z_{i,j,k}^{P}\right),i=0,1,\ldots ,\frac{n}{2},j=0,\ldots ,\frac{m}{2},k\ldots 1,\ldots ,p,$$

(A6) $$x_{i,j,k}^{P}=\left\{\begin{array}{l} \frac{i}{n}\left(L_{E}+z_{k}\frac{L_{p}-L_{E}}{c}\right),\;k>0,\\ \frac{i}{n}L_{E},\;k=0, \end{array}\right.$$

(A7) $$y_{i,j,k}^{P}=\left\{\begin{array}{l} R_{k}sin\left(\frac{l_{k}}{R_{k}}\frac{2j}{m}\right),\;k>0,\\ \frac{j}{m}B_{E},\;k=0, \end{array}\right.$$

(A8) $$z_{i,j,k}^{P}=\left\{\begin{array}{l} z_{k}+R_{k}\left[1-cos\left(\frac{l_{k}}{R_{k}}\frac{2j}{m}\right)\right],\;k>0,\\ 0,\;k=0. \end{array}\right.$$

The subscript *i* in the Formulas indicated the number of the point towards the *x* axis, the subscript *j*—towards the *y* axis, and the subscript *k*—towards the *z* axis. The established denotations and numeration of the nodes were identical for each quadrant of the model that was sectioned by two intersecting planes of *x*-*z* and *y*-*z* symmetries (cf. Figure 1c).

The next step was to establish the coordinates of points $Q_{i,j,k}$—the ends of the theoretical current line sections in the particular solid elements of the model, on the basis of the coordinates (A6)–(A8) from the relationships (A9)–(A12)

(A9) $$Q_{i,j,k}\left(x_{i,j,k}^{Q},y_{i,j,k}^{Q},z_{i,j,k}^{Q}\right),i=1,2,\ldots ,\frac{n}{2},j=1,2,\ldots ,\frac{m}{2},\;k=0,1,\ldots ,p,$$

(A10) $$x_{i-1,j-1,k}^{Q}=x_{i-1,j-1,k}^{P}+\frac{x_{i,j,k}^{P}-x_{i-1,j-1,k}^{P}}{2},$$

(A11) $$y_{i-1,j-1,k}^{Q}=y_{i-1,j-1,k}^{P}+\frac{y_{i,j,k}^{P}-y_{i-1,j-1,k}^{P}}{2},$$

(A12) $$z_{i-1,j-1,k}^{Q}=z_{i-1,j-1,k}^{P}+\frac{z_{i,j,k}^{P}-z_{i-1,j-1,k}^{P}}{2}.$$

The final step of creating the grid of points from the Relationships (A13)–(A16) on the basis of the Formulas (A10)–(A12) was to establish coordinates of the points $C_{i,j,k}$ that served as centres of segments of the theoretical current lines

(A13) $$C_{i,j,k}\left(x_{i,j,k}^{C},y_{i,j,k}^{C},z_{i,j,k}^{C}\right),i=1,2,\ldots ,\frac{n}{2},j=1,2,\ldots ,\frac{m}{2},k=1,2,\ldots ,p,$$

(A14) $$x_{i-1,j-1,k-1}^{C}=x_{i-1,j-1,k-1}^{Q}+\frac{x_{i-1,j-1,k}^{Q}-x_{i-1,j-1,k-1}^{Q}}{2},$$

(A15) $$y_{i-1,j-1,k-1}^{C}=y_{i-1,j-1,k-1}^{Q}+\frac{y_{i-1,j-1,k}^{Q}-y_{i-1,j-1,k-1}^{Q}}{2},$$

(A16) $$z_{i-1,j-1,k-1}^{C}=z_{i-1,j-1,k-1}^{Q}+\frac{z_{i-1,j-1,k}^{Q}-z_{i-1,j-1,k-1}^{Q}}{2}.$$

Finally, the grid of points representing the electrically conductive area of concrete under the alternating current polarization, which was between the counter electrode and the working electrode, and was described with the above Formulas, was characterized by “flexibility” in adjusting to varying sizes of the rectangular counter electrode, the diameter and length of the rebar polarized area, and a thickness of concrete cover. A reference comparison of changes in the grid shape at changed sizes of the counter electrode is illustrated in Figure A2.

## Appendix B

Based on the *3D model* assumptions, which were presented in Section 2, the impact of moisture content in concrete on the shapes of impedance spectra of reinforcing steel in concrete elements is expressed by the coefficient $\psi_{i,j,k}$ given by the Formula (5). It is a parameter of local coefficient for concrete geometry $\gamma_{i,j}^{c}$ expressed by the Formula (1). The bulk density of concrete $\rho^{c}$, present in this Formula, was determined for the independent concrete samples with dimensions of 50 × 100 × 100 mm, formed simultaneously with the base elements. For concrete of series *S1* (without any additives), $\rho^{c}$ = 2.25 ± 0.02 g/cm^3^, whereas for concrete of series *S2* (with chlorides) $\rho^{c}$ = 2.22 ± 0.01 g/cm^3^.

Mass moisture in concrete $w_{i,j,k}$ of each solid element with a volume $V_{i,j,k}$, present in the Formula (5), can be theoretically determined from the spatial distribution of moisture content, the measurement of which cannot be performed due to technical reasons—cf. [65]. Therefore, the simplified approach was adopted and moisture content in each point $C_{i,j,k}\left(x_{i,j,k}^{C},y_{i,j,k}^{C},z_{i,j,k}^{C}\right)$ (Figure 1d) was approximated with average moisture content by taking $w_{i,j,k}=w_{mid}$. For that purpose, moisture content was measured by the dielectric method using the moisture meter WIP-24. Measurements were taken in 9 uniformly arranged points on top surfaces of the test elements. The measurement range of the device was 5–6 cm, which means that average moisture content in concrete could be identified for the whole electrically conductive area under polarization. The measured dielectric moisture content in concrete was converted into mass moisture using the empirical formulas shown in Figure A3. The empirical relationships in the form of logarithmic regression equations were obtained from the correlation tests on the above additional concrete test elements with a dimension of 50 × 100 × 100 m After saturation with water, cyclic tests on moisture content in concrete were performed during drying under natural conditions using simultaneously two methods: the drying and weighing method, and the dielectric method—cf. [65]. The results obtained for concrete of series *S1* and *S2* are presented as diagrams in Figure A3a,b.

## Appendix C

**Table A1:** Electrochemical parameters of initial spectra, introduced into Formulas for the *3D model* to simulate the effect of a varying width $B_{E}$ of the counter electrode and statistical evaluation of matching the model spectrum to the empirical one.

Parameters	Series *S1* Spectra *P1_B_*, *M1_B_*		Series *S2* Spectra *P2_B_*, *M2_B_*	
$R^{1}$	0	Ω	0	Ω
$R^{2}$	1.080	kΩ	0.682	kΩ
$R^{2a}$	1.080	kΩ	0.697	kΩ
$Y^{2}$	100.5	nF⋅s^α−1^	214.7	nF∙s^α−1^
$\alpha^{2}$	0.668		0.699	
$Y^{2a}$	100.5	nF⋅s^α−1^	239.9	nF∙s^α−1^
$\alpha^{2a}$	0.768		0.683	
$Y^{3}$	1967	μF⋅s^α−1^	653.0	mF∙s^α−1^
$\alpha^{3}$	0.203		0.843	
$Y^{0}$	7445	μF⋅s^α−1^	2818	μF∙s^α−1^
$\alpha^{0}$	0.827		0.302	
$R^{t}$	4.642	kΩ	0.682	kΩ
a	1	%	5	%
$\chi^{2}$	450.3		274.6	
q	1.73		1.35	

**Table A2:** Electrochemical parameters of initial spectra, introduced into Formulas for the *3D model* to simulate the effect of a varying length $L_{E}$ of the counter electrode and statistical evaluation of matching the model spectrum to the empirical one.

Parameters	Series *S1* Spectra *P1_L_*, *M1_L_*		Series *S2* Spectra *P4_L_*, *M4_L_*	
$R^{1}$	0	Ω	0	Ω
$R^{2}$	2.044	kΩ	0.391	kΩ
$R^{2a}$	2.343	kΩ	1.912	kΩ
$Y^{2}$	5.855	nF∙s^α−1^	3.802	nF∙s^α−1^
$\alpha^{2}$	0.931		0.888	
$Y^{2a}$	132.3	nF∙s^α−1^	26.37	nF∙s^α−1^
$\alpha^{2a}$	0.674		0.776	
$Y^{3}$	6075	μF∙s^α−1^	14070	μF∙s^α−1^
$\alpha^{3}$	0.556		0.333	
$Y^{0}$	1021	μF∙s^α−1^	95.63	μF∙s^α−1^
$\alpha^{0}$	0.153		0.444	
$R^{t}$	1.203	kΩ	0.331	kΩ
a	1	%	1	%
$\chi^{2}$	194.1		138.1	
q	1.14		0.96	

**Table A3:** Comparison of electrochemical parameters of the electrical equivalent circuit from Figure 7, determined in the first stage of iterative determination of polarization range of reinforcement in concrete for two lengths $L_{E}$ of the rectangular counter electrodes.

Parameters	$L_{E}=150\;\mathrm{mm}$		$L_{E}=50\;\mathrm{mm}$	
$R^{1}$	0.004	Ω	0.017	Ω
$R^{2}$	962.7	Ω	1612	Ω
$R^{2a}$	740.5	Ω	2012	Ω
$Y^{2}$	0.403	nF∙s^α−1^	0.298	nF∙s^α−1^
$\alpha^{2}$	0.988		0.960	
$Y^{2a}$	42.48	nF∙s^α−1^	18.95	nF∙s^α−1^
$\alpha^{2a}$	0.793		0.799	
$Y^{3}$	3149	μF∙s^α−1^	2186	μF∙s^α−1^
$\alpha^{3}$	0.435		0.376	
$Y^{0}$	46.67	μF∙s^α−1^	0.153	μF∙s^α−1^
$\alpha^{0}$	0.494		0.836	
$R^{t}$	196.7	Ω	887.3	Ω
a	2	%	2	%
$\chi^{2}$	6.20		56.57	
q	0.31		0.93	
